# Novel machine‐learning prediction tools for overall survival of patients with chondrosarcoma: Based on recursive partitioning analysis

**DOI:** 10.1002/cam4.70058

**Published:** 2024-08-09

**Authors:** Xiong‐Gang Yang, Shan‐Shan Yang, Yi Bao, Qi‐Yang Wang, Zhi Peng, Sheng Lu

**Affiliations:** ^1^ Department of Orthopedics, The First People's Hospital of Yunnan Province The Affiliated Hospital of Kunming University of Science and Technology Kunming Yunnan China; ^2^ The Key Laboratory of Digital Orthopedics of Yunnan Province Kunming Yunnan China; ^3^ Department of Prosthodontics Affiliated Stomatological Hospital of Zunyi Medical University, Zunyi Medical University Zunyi China

**Keywords:** C5.0 algorithm, chondrosarcoma, decision tree model, overall survival, recursive partitioning analysis

## Abstract

**Background:**

Chondrosarcoma (CHS), a bone malignancy, poses a significant challenge due to its heterogeneous nature and resistance to conventional treatments. There is a clear need for advanced prognostic instruments that can integrate multiple prognostic factors to deliver personalized survival predictions for individual patients. This study aimed to develop a novel prediction tool based on recursive partitioning analysis (RPA) to improve the estimation of overall survival for patients with CHS.

**Methods:**

Data from the Surveillance, Epidemiology, and End Results (SEER) database were analyzed, including demographic, clinical, and treatment details of patients diagnosed between 2000 and 2018. Using C5.0 algorithm, decision trees were created to predict survival probabilities at 12, 24, 60, and 120 months. The performance of the models was assessed through confusion scatter plot, accuracy rate, receiver operator characteristic (ROC) curve, and area under ROC curve (AUC).

**Results:**

The study identified tumor histology, surgery, age, visceral (brain/liver/lung) metastasis, chemotherapy, tumor grade, and sex as critical predictors. Decision trees revealed distinct patterns for survival prediction at each time point. The models showed high accuracy (82.40%–89.09% in training group, and 82.16%–88.74% in test group) and discriminatory power (AUC: 0.806–0.894 in training group, and 0.808–0.882 in test group) in both training and testing datasets. An interactive web‐based shiny APP (URL: https://yangxg1209.shinyapps.io/chondrosarcoma_survival_prediction/) was developed, simplifying the survival prediction process for clinicians.

**Conclusions:**

This study successfully employed RPA to develop a user‐friendly tool for personalized survival predictions in CHS. The decision tree models demonstrated robust predictive capabilities, with the interactive application facilitating clinical decision‐making. Future prospective studies are recommended to validate these findings and further refine the predictive model.

## 1 INTRODUCTION

Chondrosarcoma, a malignant bone tumor characterized by the formation of cartilaginous matrix without typical bone formation, is known to affect individuals across various age groups, with a higher incidence observed in the elderly population.^1^ With an annual incidence of five cases per million, chondrosarcoma represents 30% of all malignant bone tumors, ranking second only to osteosarcoma.^2^, ^3^, ^4^ In comparison to Ewing sarcoma and osteosarcoma, chondrosarcoma is considered a less aggressive form of cancer.^5^ It most commonly affects the pelvis and proximal femur, posing significant challenges due to its location and aggressive behavior.^6^, ^7^ The cornerstone of chondrosarcoma treatment lies in surgical resection, as these tumors typically demonstrate resistance to conventional‐dose radiotherapy and chemotherapy.^8^, ^9^, ^10^ This resistance is largely attributed to the rich extracellular matrix, limited vascularization, less cellular dividing, and sluggish growth characteristics of chondrosarcoma.^11^

The prognosis for patients with chondrosarcoma varies significantly and is largely contingent upon factors such as tumor grade, size, location, and other patient‐specific characteristics. Therapeutic strategies diverge considerably across various tumor grades. Low‐grade chondrosarcomas, with their lower risk of local recurrence and distant metastasis, are associated with increased overall survival and can often be effectively managed through curettage. Conversely, higher‐grade tumors carry a greater likelihood of local recurrence and metastasis, leading to a reduced overall survival, necessitating more extensive surgical excision or even amputation in some cases.^12^, ^13^ While histological grading is a crucial determinant of patient survival, it is subjective and can vary among pathologists, underscoring the urgency for more objective and reliable indicators of overall survival. Identifying independent prognostic factors that influence survival through extensive patient cohort studies holds considerable significance for both patients and clinicians, offering insights into future prognoses, guiding the selection of optimal treatment strategies, and determining the extent of surgical intervention. Over the years, numerous retrospective studies have explored the prognostic significance of various clinicopathological variables in chondrosarcoma.^14^, ^15^, ^16^, ^17^, ^18^, ^19^ Among the identified prognostic factors, there is a clear need for advanced prognostic instruments that can integrate multiple variables to deliver personalized survival predictions for individual patients.

Clinical prediction models, which combine biological, clinical, and statistical methods to estimate the risk of an outcome, offer a promising solution to this challenge.^20^, ^21^, ^22^ In the context of chondrosarcoma, many prediction models have been proposed, primarily based on logistic regression or Cox proportional hazards regression. These studies often visualize regression results through nomograms, graphical representations that assign point values to individual predictors, allowing for the calculation of total points and subsequent estimation of survival probabilities at several time points.^14^, ^15^, ^16^, ^18^, ^19^ When utilizing these prediction models, users must manually score each predictor before summing the scores to estimate overall survival.

Recursive partitioning analysis (RPA) and decision tree models have emerged as attractive alternatives to traditional statistical approaches.^23^ RPA is a nonparametric method that recursively splits the patient population into subgroups based on optimal cutoff values. This results in a series of nested partitions or nodes, each representing a distinct patient subgroup with homogeneous outcomes. Decision trees, constructed using RPA, visually represent the relationships between predictors and outcomes, providing a straightforward and intuitive tool for clinical decision support.^24^ The RPA and decision tree models can accommodate complex interactions and produce easily interpretable models that are accessible to clinicians without specialized statistical training. Additionally, they can be readily implemented in software and web‐based platforms, facilitating integration into clinical workflows and improving accessibility to a wide range of healthcare providers. The application of RPA and decision tree models in the field of chondrosarcoma is still in its infancy.

Therefore, by leveraging the strengths of RPA and decision tree modeling, we aimed to create a user‐friendly tool that integrates multiple prognostic factors to generate personalized survival predictions for patients with chondrosarcoma. Furthermore, we planned to translate this model into an interactive software application and web‐based platform, making it widely available to clinicians as a decision aid in selecting appropriate treatment strategies for their patients with chondrosarcoma.

## 2 MATERIALS AND METHODS

This investigation adhered to the principles outlined in the Transparent Reporting of a Multivariable Prediction Model for Individual Prognosis or Diagnosis (TRIPOD) statement to ensure the clarity and reproducibility of our research findings.

### 2.1 Data source

The dataset for our analysis was sourced from the Surveillance, Epidemiology, and End Results (SEER) database, a nationwide cancer registry system administered by the National Cancer Institute (NCI) in the US Our study encompassed individuals diagnosed with chondrosarcoma, with pertinent data meticulously extracted from the registry. Key data items included patient demographics, clinical features, therapeutic interventions, and longitudinal survival information.

### 2.2 Inclusion and exclusion criteria

Records retrieved from the SEER database had to meet the following inclusion criteria: (1) a definitive diagnosis of chondrosarcoma, as evidenced by International Classification of Diseases for Oncology, 3rd edition (ICD‐O‐3) codes; (2) diagnoses made within the period extending from 2000 to 2018; and (3) availability of comprehensive data regarding baseline characteristics, tumor histology, tumor grade, therapeutic modalities, and clear survival status at each time point (12, 24, 60, and 120 months).

Patients would be non‐eligible for inclusion under the following conditions: (1) diagnosed predating the year 2000; (2) inaccuracies or ambiguities concerning the duration of survival; and (3) records flagged as censored during the follow‐up interval, indicating incomplete survival data.

### 2.3 Collected variables and data processing

Prognostic factors used for analysis included: (1) age of diagnosis; (2) race (white, black, or others); (3) sex (male or female); (4) year of diagnosis (<2010 or ≥2010); (5) histology type (chondrosarcoma‐NOS, dedifferentiated chondrosarcoma, mesenchymal chondeosarcoma, myxoid chondrosarcoma, juxtacortical chondrosarcoma, clear cell chondrosarcoma, or malignant chondroblastoma); (6) tumor grade (Grade I: well differentiated; Grade II: moderately differentiated; Grade III: poorly differentiated; Grade IV: undifferentiated); (7) surgery procedure (performed, recommended but not performed, or not recommended); (8) radiotherapy (yes or no); (9) chemotherapy (yes or no); (10) regional lymph node surgery (yes or no); (11) systematic therapy (yes or no); (12) number of in situ malignant tumors (single or multiple); (13) metastasis to bone (yes or no); (14) visceral metastasis to brain/liver/lung (yes or no); (15) tumor size.

**FIGURE 1:**
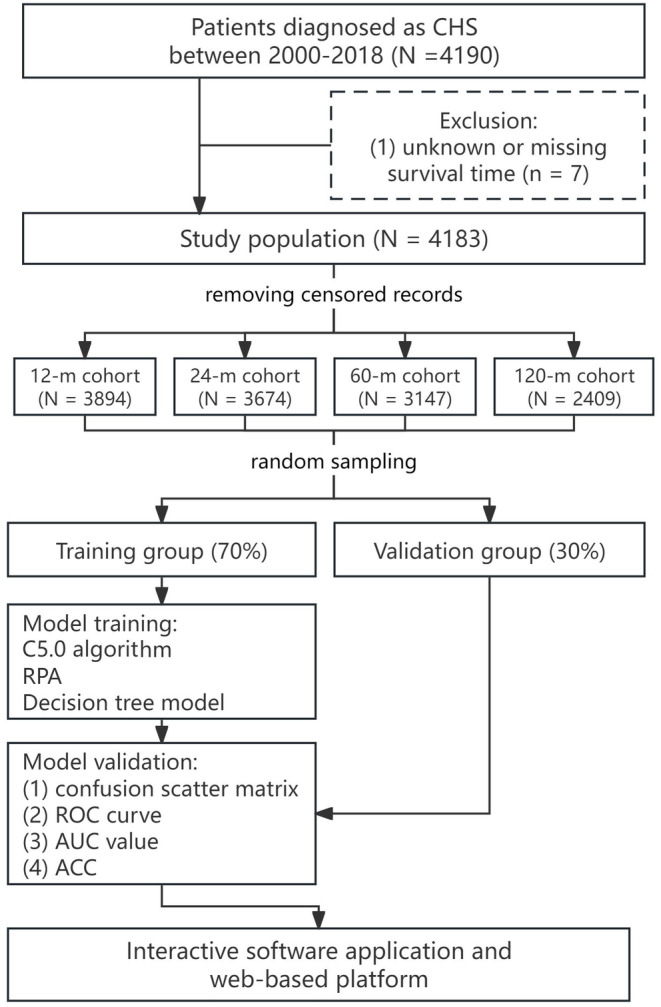
The flow chart of the study design. ACC, accuracy rate; AUC, area under ROC curve; CHS, chondrosarcoma; ROC, receiver operator characteristic; RPA, recursive partitioning analysis.

The primary outcome in this cohort study was the patients' survival statuses (alive or dead) at 12, 24, 60, and 120 months. After removing the records censored during follow‐up and those without comprehensive information about survival, a total of 3894, 3674, 3147, and 2409 patients were included for the four cohorts (as shown in the flowchart in Figure 1).

### 2.4 Statistical analyses

**FIGURE 2:**
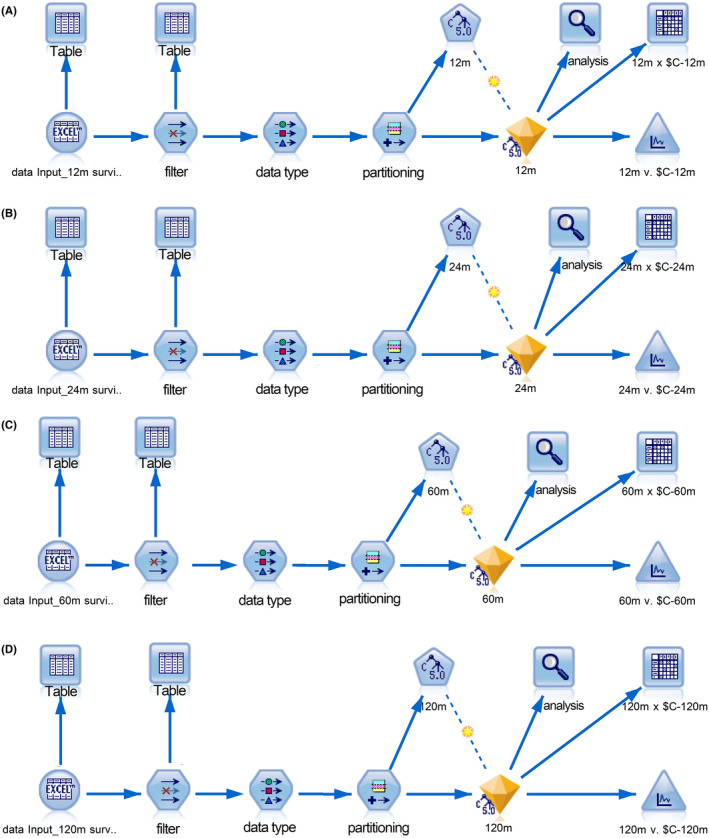
The screenshot captures the comprehensive analysis flow within SPSS Modeler, detailing each step from initial data loading through to final model validation. Data undergoes rigorous screening and classification before being subjected to the C5.0 algorithm, which constructs dichotomous decision trees tailored to predict survival at 12, 24, 60, and 120 months.

Continuous variables such as age and tumor size were reported as mean ± standard deviation (SD), while other variables were expressed as percentages. Following the exclusion of censored records, four distinct cohorts were established. These cohorts were subsequently partitioned into training and test datasets with a randomized split ratio of 7:3, facilitated by a computational algorithm. For the purpose of predicting survival statuses at specific time points (12, 24, 60, and 120 months), dichotomous decision trees were crafted. This was achieved using the C5.0 algorithm, which is integrated within the SPSS Modeler version 18.0 software (IBM Corp., Armonk, New York, USA). A visual representation of the analytical workflow, encompassing stages from data importation to model training and validation, is depicted in Figure 2.

In order to assess the performances of the newly developed decision trees, we employed the confusion scatter matrix and ROC curves, from which we computed the accuracy rate (ACC) and the area under the ROC curve (AUC). Model validation processes were executed in both the training and test datasets. Subsequently, these models were adapted into an interactive software tool and a web‐based platform through the utilization of the “shiny” package within R version 4.3.3 (Foundation for Statistical Computing, Vienna, Austria). The above model validation process was carried out using R program, with a statistical significance threshold set at less than 0.05.

## 3 RESULTS

### 3.1 Baseline characteristics

**FIGURE 3:**
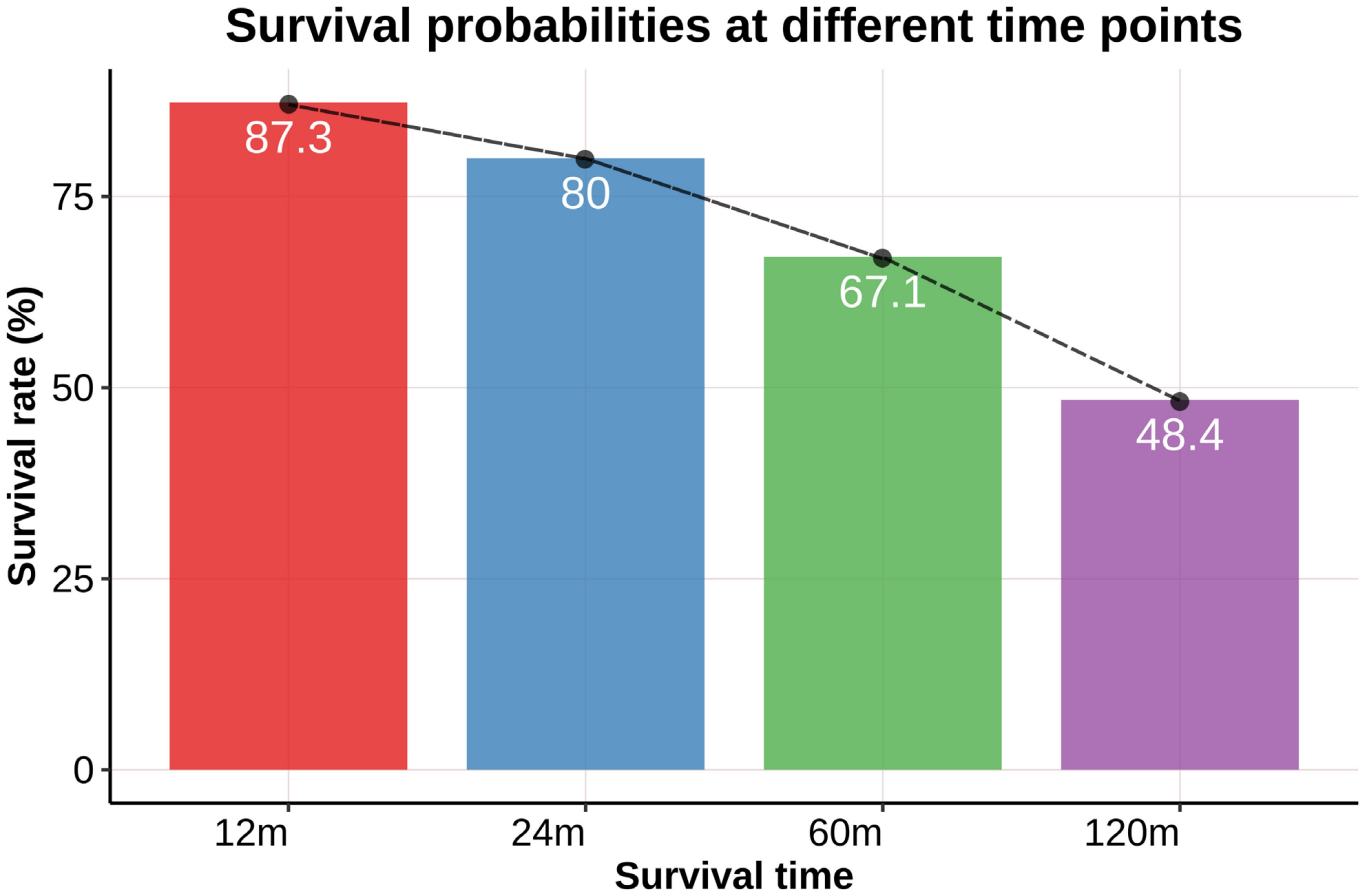
The survival probabilities for the four cohorts at 12 (87.3%), 24 (80.0%), 60 (67.1%), and 120 (48.4%) months.

Consecutive cohorts of 3894, 3674, 3147, and 2409 patients with definitive survival status were included for the 12‐, 24‐, 60‐, and 120‐month analyses, respectively. Table 1 presents the baseline characteristics of the included patients. Among the 12‐month cohort, the mean age was 52.8 ± 18.5 years; the male gender accounted for 54.3% of the population; the mean tumor size measured 82.8 ± 55.9 mm; surgery was performed in 85.3% of the patients, whereas 14.5% and 8.1% received radiotherapy and chemotherapy, respectively; metastatic events were documented in 0.8% of patients involving bone and 3.0% affecting the brain, liver, or lung. Survival probabilities at 12, 24, 60, and 120 months were recorded as 87.3%, 80.0%, 67.1%, and 48.4%, respectively (as depicted in Figure 3).

**TABLE 1:** Baseline characteristics of the included patients for four different time points.

Variables	12 months (*n* = 3894)	24 months (*n* = 3674)	60 months (*n* = 3147)	120 months (*n* = 2409)
Survival status				
Alive	3399 (87.3%)	2939 (80.0%)	2112 (67.1%)	1166 (48.4%)
Dead	495 (12.7%)	735 (20.0%)	1035 (32.9%)	1243 (51.6%)
Age (years)				
Mean (SD)	52.8 (18.5)	52.8 (18.6)	53.3 (18.6)	54.9 (18.7)
Sex				
Male	2116 (54.3%)	2002 (54.5%)	1726 (54.8%)	1334 (55.4%)
Female	1778 (45.7%)	1672 (45.5%)	1421 (45.2%)	1075 (44.6%)
Race				
White	3368 (86.5%)	3182 (86.6%)	2739 (87.0%)	2111 (87.6%)
Black	283 (7.3%)	265 (7.2%)	225 (7.1%)	164 (6.8%)
Others	243 (6.3%)	227 (6.2%)	183 (5.8%)	134 (5.5%)
Year of diagnosis				
<2010	2092 (53.7%)	2086 (56.8%)	2069 (65.7%)	1893 (78.6%)
≥2010	1802 (46.3%)	1588 (43.2%)	1078 (34.3%)	516 (21.4%)
Histology type				
CHS, NOS	3144 (80.7%)	2954 (80.4%)	2507 (79.7%)	1865 (77.4%)
Dedifferentiated CHS	314 (8.1%)	299 (8.1%)	284 (9.0%)	264 (11.0%)
Mesenchymal CHS	61 (1.6%)	57 (1.5%)	49 (1.6%)	41 (1.7%)
Myxoid CHS	236 (6.1%)	288 (6.25)	196 (6.2%)	160 (6.6%)
Juxtacortical CHS	41 (1.1%)	41 (1.1%)	35 (1.1%)	25 (1.0%)
Clear cell CHS	64 (1.6%)	61 (1.6%)	48 (1.5%)	38 (1.6%)
Chondroblastoma, malignant	34 (0.9%)	34 (0.9%)	28 (0.9%)	16 (0.7%)
Tumor grade				
Grade I	1203 (30.9%)	1123 (30.6%)	953 (30.3%)	675 (28.0%)
Grade II	1378 (35.4%)	1304 (35.5%)	1105 (35.1%)	816 (33.9%)
Grade III	437 (11.2%)	409 (11.1%)	353 (11.2%)	292 (12.1%)
Grade IV	291 (7.5%)	285 (7.8%)	263 (8.4%)	246 (10.2%)
Unknown	585 (15.0%)	553 (15.1%)	473 (15.0%)	380 (15.8%)
Tumor size (mm)				
Mean (SD)	82.8 (55.9)	88.6 (59.9)	117.3 (58.7)	117.3 (58.7)
Surgery procedures				
Not recommended	426 (10.9%)	406 (11.1%)	367 (11.7%)	322 (13.4%)
Performed	3322 (85.3%)	3128 (85.1%)	2655 (84.4%)	1971 (81.8%)
Recommended but not performed	110 (2.8%)	105 (2.9%)	95 (3.0%)	90 (3.7%)
Unknown	36 (0.9%)	35 (1.0%)	30 (1.0%)	26 (1.1%)
Radiotherapy				
None/Unknown	3330 (85.5%)	3135 (85.3%)	2682 (85.3%)	2027 (84.2%)
Yes	564 (14.5%)	539 (14.7%)	465 (14.8%)	382 (15.9%)
Chemotherapy				
None/Unknown	3577 (91.9%)	3370 (91.7%)	2865 (91.0%)	2149 (89.2%)
Yes	317 (8.1%)	304 (8.3%)	282 (9.0%)	260 (10.8%)
Regional LN surgery				
Yes	164 (4.2%)	147 (4.0%)	124 (3.9%)	91 (3.8%)
No	3028 (77.8%)	2832 (77.1%)	2346 (74.5%)	1654 (68.7%)
Unknown	702 (18.0%)	695 (18.9%)	677 (21.5%)	664 (27.6%)
Systemic therapy				
No	2389 (61.4%)	2183 (59.4%)	1684 (53.5%)	981 (40.7%)
Yes	164 (4.2%)	153 (4.2%)	132 (4.2%)	114 (4.7%)
Unknown	1341 (34.4%)	1338 (36.4%)	1331 (42.3%)	1314 (54.5%)
Number of in situ malignant tumors				
Single	3037 (78.0%)	2862 (77.9%)	2413 (76.7%)	1808 (75.1%)
Multiple	857 (22.0%)	812 (22.1%)	734 (23.3%)	601 (24.9%)
Metastasis to bone				
No	1718 (44.1%)	1510 (41.1%)	1019 (32.4%)	473 (19.6%)
Yes	31 (0.8%)	29 (0.8%)	26 (0.8%)	25 (1.0%)
Unknown	2145 (55.1%)	2135 (58.1%)	2102 (66.8%)	1911 (79.3%)
Metastasis to brain/liver/lung				
No	1632 (41.9%)	1428 (38.9%)	937 (29.8%)	395 (16.4%)
Yes	115 (3.0%)	109 (3.0%)	106 (3.4%)	101 (4.2%)
Unknown	2147 (55.1%)	2137 (58.2%)	2104 (66.9%)	1913 (79.4%)

Abbreviations: CHS, chondrosarcoma; Grade I, well differentiated; Grade II, moderately differentiated; Grade III, poorly differentiated; Grade IV, undifferentiated; LN, lymph node; SD, standard deviation.

### 3.2 Development and validation of the decision tree models

**FIGURE 4:**
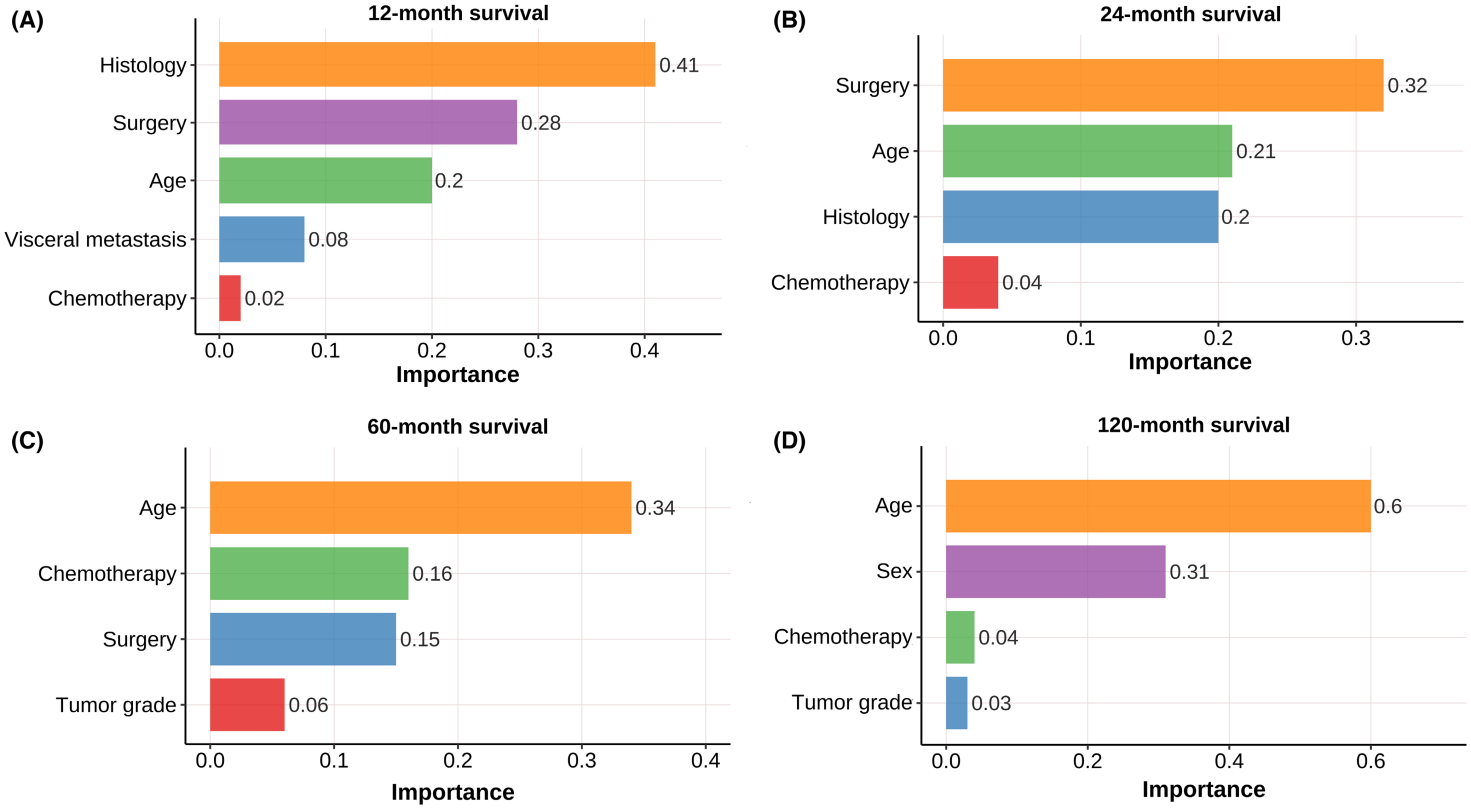
The hierarchy of significance among predictors within the decision tree framework is elucidated at distinct time intervals: 12 (A), 24 (B), 60 (C), and 120 months (D). This ranking reflects the relative contribution of each variable in determining survival probability at varying stages.

**FIGURE 5:**
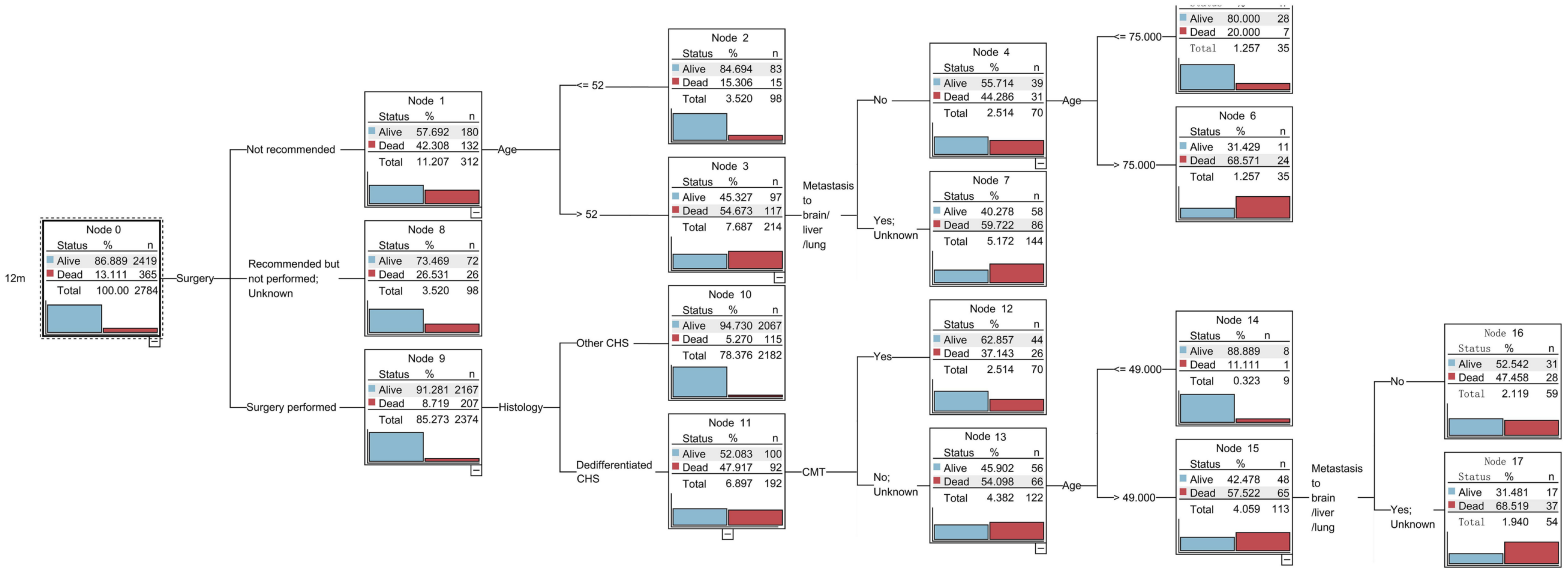
Decision tree model for 12‐month survival status. CMT, chemotherapy; CHS, chondrosarcoma.

**FIGURE 6:**
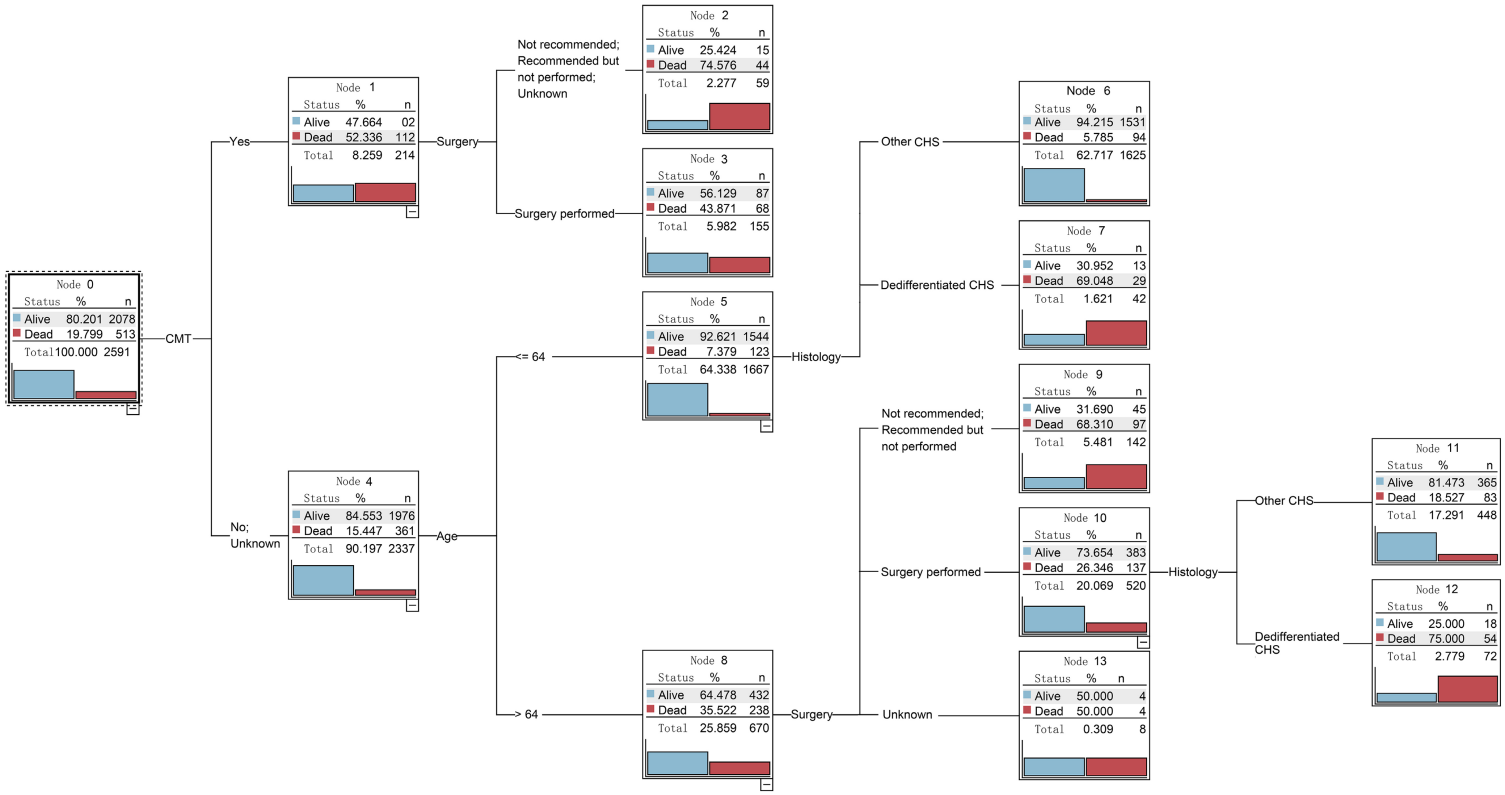
Decision tree model for 24‐month survival status. CHS, chondrosarcoma; CMT, chemotherapy.

**FIGURE 7:**
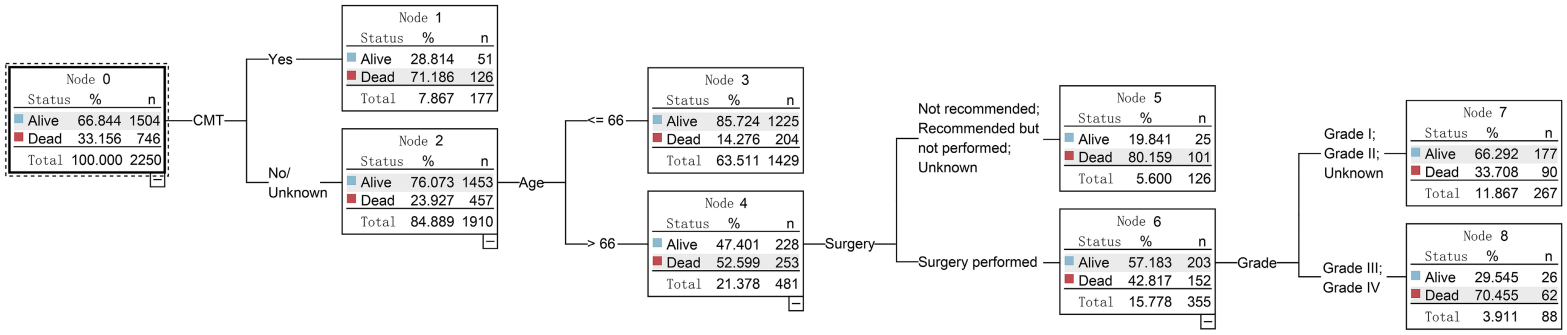
Decision tree model for 60‐month survival status. CMT, chemotherapy.

**FIGURE 8:**
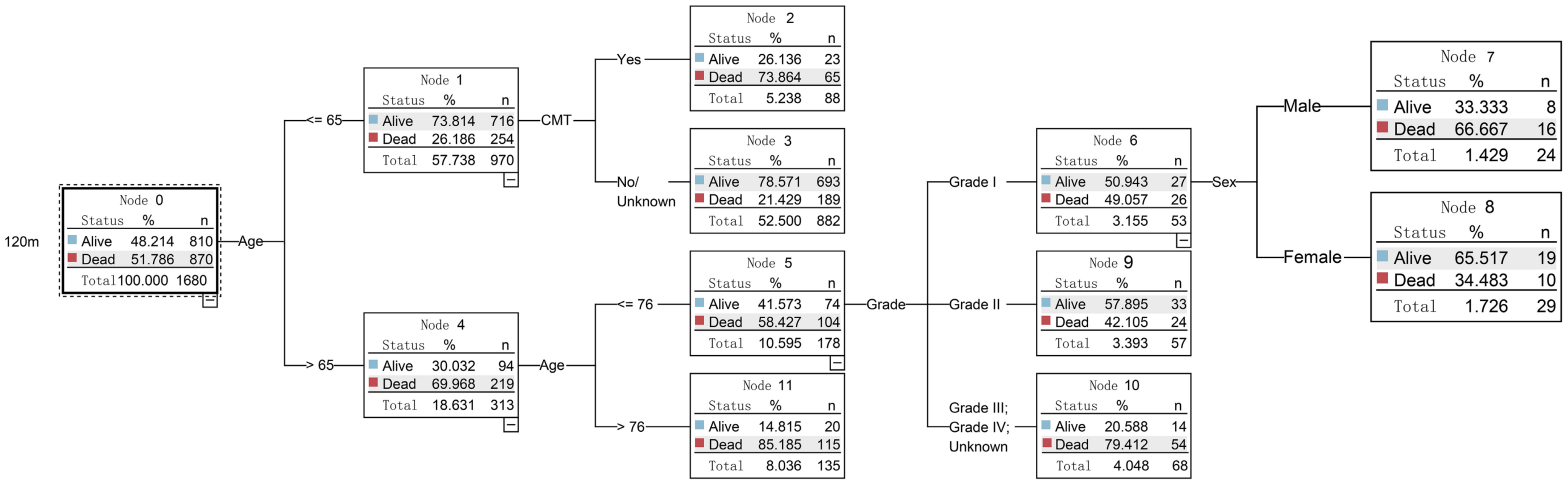
Decision tree model for 120‐month survival status. CMT, chemotherapy.

Leveraging the C5.0 algorithm, we pinpointed the most influential prognostic indicators, which encompassed tumor histology, surgery procedure, age, visceral metastasis to brain/liver/lung, chemotherapy, tumor grade, and sex. The rankings of importance of the predictors for the four cohorts are illustrated in Figure 4. These prognostic markers served as the foundation for constructing decision tree models designed to forecast survival status at 12, 24, 60, and 120 months. The visual representations of the decision trees are presented in Figures 5, 6, 7, 8. The depth of the decision tree was limited within five layers. In the 12‐month model, surgery procedure, age, histology type, visceral metastasis to brain/liver/lung, and chemotherapy emerged as key determinants for prediction; in the 24‐month model, chemotherapy, surgery procedure, age, and histology type were included; in the 60‐month model, chemotherapy, age, surgery procedure, and tumor grade were included; in the 120‐month model, age, chemotherapy, tumor grade, and sex were included. These graphical models empower clinicians to swiftly ascertain predicted survival rates at varying time intervals.

**FIGURE 9:**
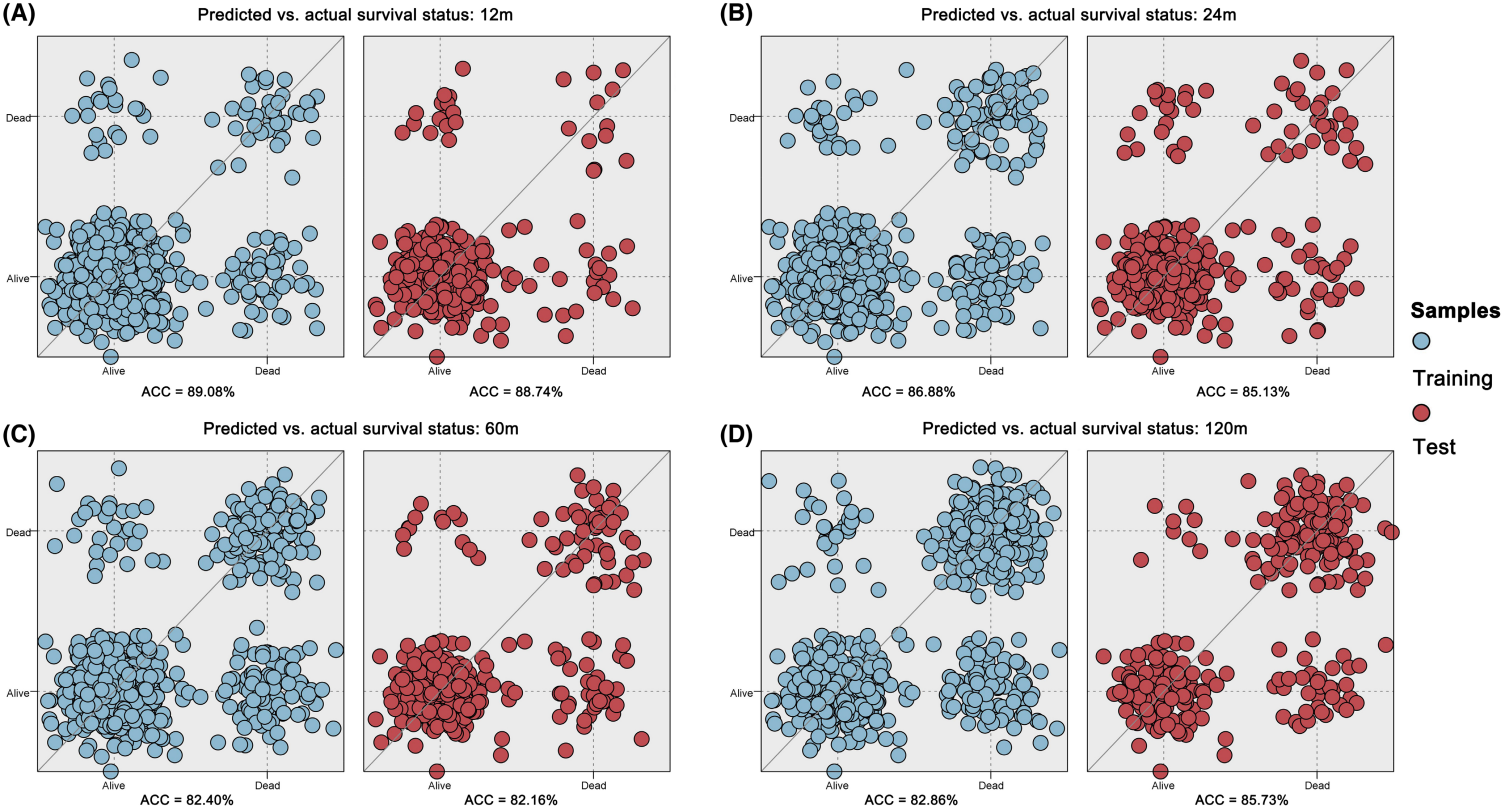
Confusion scatter diagrams elucidate the agreement between predicted and observed survival outcomes at 12 (A), 24 (B), 60 (C), and 120 months (D) for both the training and testing groups. Each model exhibited commendable concordance between the predicted and actual survival states. The accuracy rate (ACC) serves as an indicator of the overall predictive performance, confirming the efficacy of the models across different time points.

**FIGURE 10:**
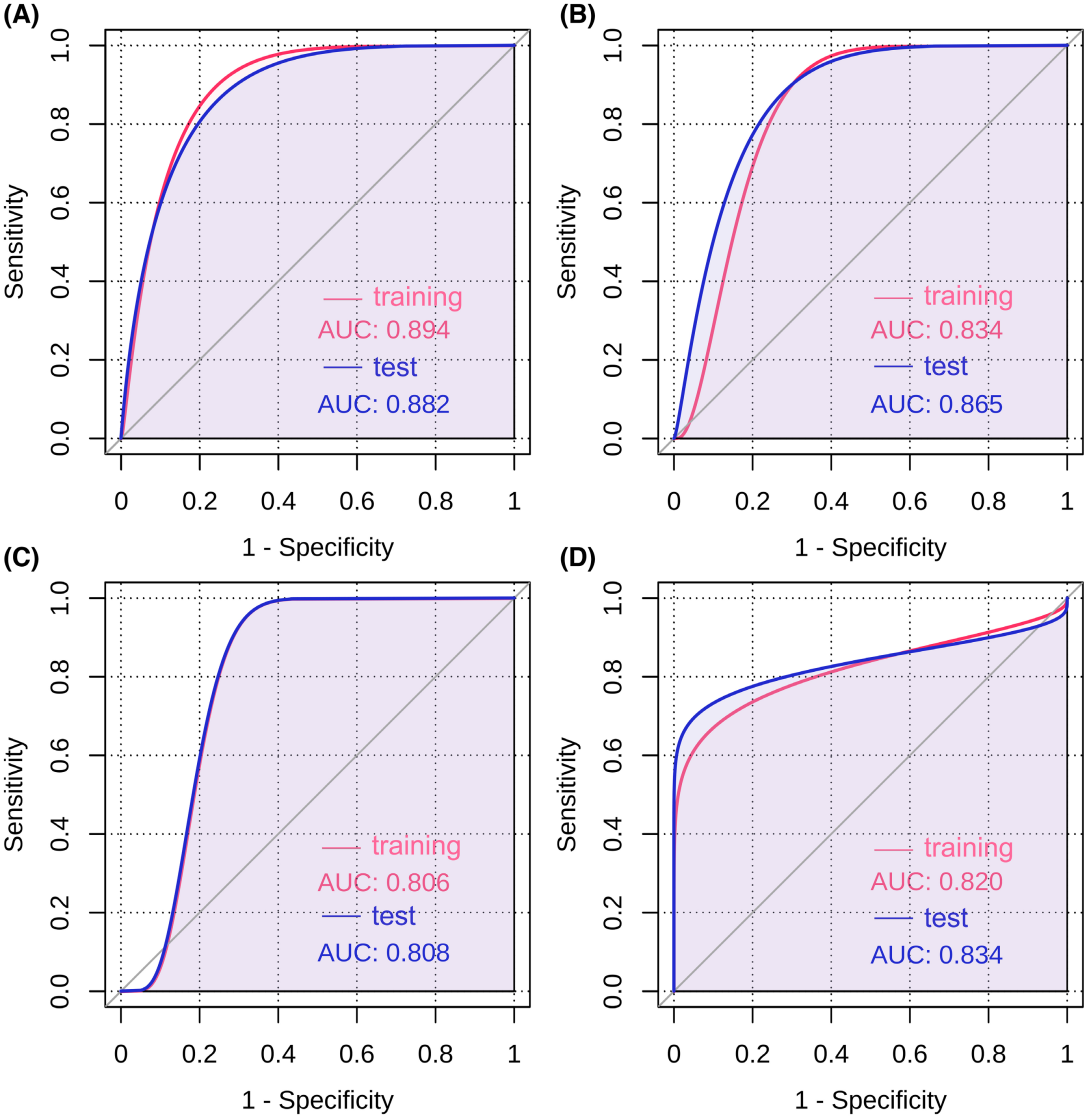
Receiver Operating Characteristic (ROC) curves illustrate the performance of the decision tree models at distinct time points: 12 (A), 24 (B), 60 (C), and 120 months (D), with separate analyses conducted for both the training and test datasets. The area under the ROC curve (AUC), a metric quantifying the discriminative ability of the models, was found to be 0.894, 0.834, 0.806, and 0.820 for the training set, and 0.882, 0.865, 0.808, and 0.834 for the test set, respectively. These figures reflect the robustness and reliability of the models in predicting outcomes at various follow‐up durations.

Figure 9 elucidates the confusion scatter diagrams that map the congruence between predicted and actual survival statuses at 12 (Figure 9A), 24 (Figure 9B), 60 (Figure 9C), and 120 (Figure 9D) months, with both training and testing datasets scrutinized. The plots exhibit a high degree of alignment between the predicted and actual survival statuses, as the majority of data points cluster closely along the diagonal line. The accuracy rates were 89.08%, 86.88%, 82.40%, and 82.86% for training group, and 88.74%, 85.13%, 82.16%, and 85.73% for test group at 12, 24, 60, and 120 months, respectively. Furthermore, Figure 10 provides the ROC curves for both training and testing datasets at 12 (Figure 10A), 24 (Figure 10B), 60 (Figure 10C), and 120 (Figure 10D) months. The AUC values were indicative of favorable discriminatory power of the models: 0.894, 0.834, 0.806, and 0.820 for the training set, and 0.882, 0.865, 0.808, and 0.831 for the testing set, underscoring the robustness and reliability of our predictive models.

### 3.3 Interactive application and web‐based platform

**FIGURE 11:**
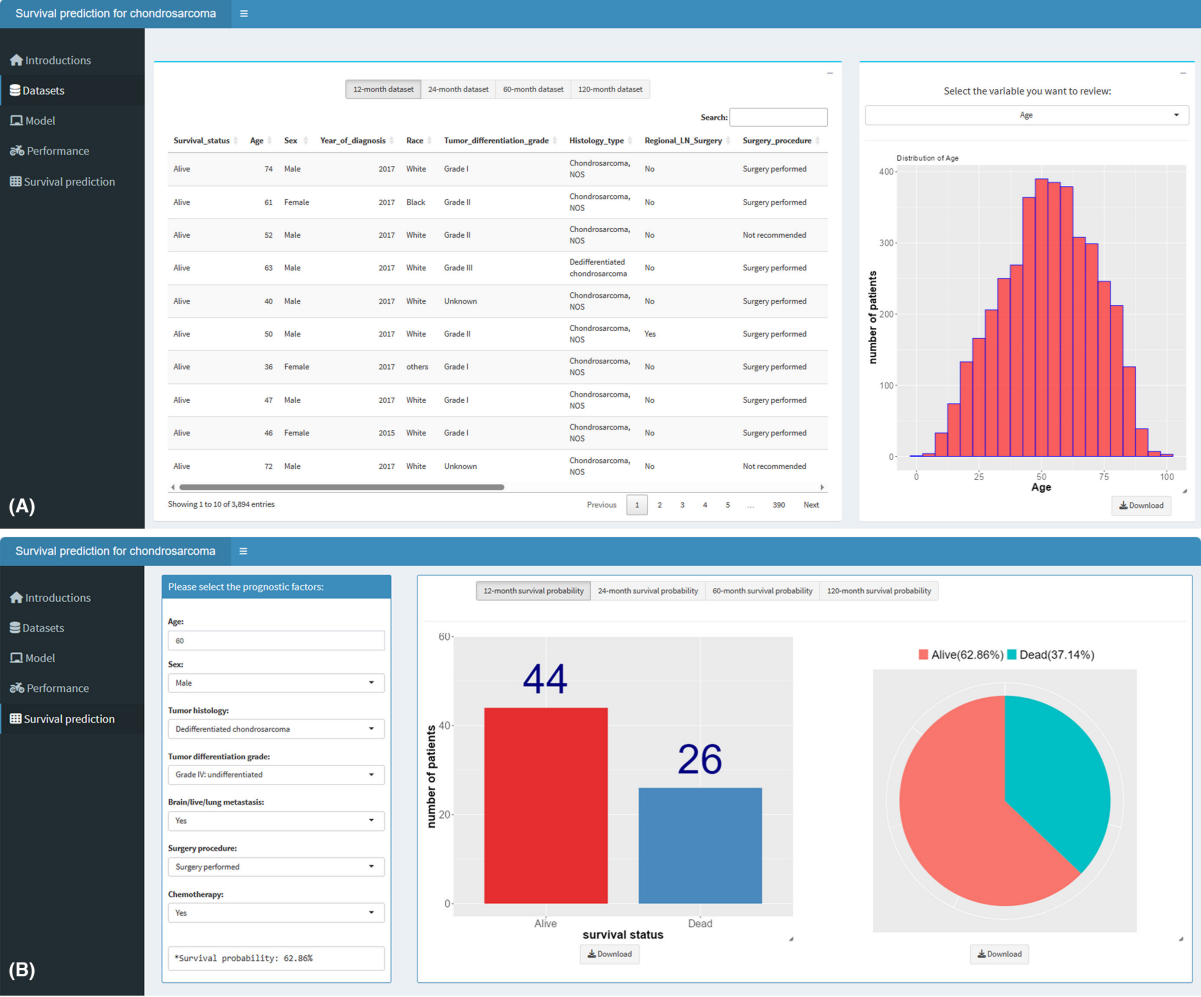
Interactive survival prediction software for chondrosarcoma patients. The software interface consists of five dedicated tab pages: “Introduction,” “Datasets,” “Model,” “Performance,” and “Survival Prediction.” Within the “Datasets” page, users are afforded the opportunity to review and perform automated, visual data analysis on the datasets pertinent to the modeling process (A). Progressing to the “Survival Prediction” page, once patient clinical characteristics have been specified, the software enables the automatic generation of projected survival probabilities at four predetermined time intervals (B).

To streamline the intricate manual computations associated with graphical decision tree models, we have enhanced our model by developing an interactive application. The application features five distinct tabs, each designed with a specific purpose: an overview of the application, detailed descriptions of the demographic characteristics of the study population, the process of constructing the model, an assessment of the performance, and an intuitive interface for predicting survival probabilities (Figure 11). Users can customize their review of the study population's demographics, scrutinize the model creation and validation processes, and interactively automate survival probability predictions across four predefined time points. When forecasting survival probabilities, users simply select the prognostic factors to receive bar and pie charts illustrating the distribution of survival statuses at four distinct time points. In an effort to promote widespread utilization and acceptance of this predictive tool, we have made it readily accessible via a web server (URL: https://yangxg1209.shinyapps.io/chondrosarcoma_survival_prediction/).

For instance, a male patient, aged 60, diagnosed with dedifferentiated (grade IV: undifferentiated) chondrosarcoma and exhibiting visceral metastasis to the brain/liver/lung, who has undergone surgical intervention and chemotherapy, is projected to have survival probabilities of 62.86%, 56.13%, 28.81%, and 26.14% at 12, 24, 60, and 120 months.

## 4 DISCUSSION

Our study has successfully identified critical prognostic indicators including tumor histology, surgery procedure, age, visceral (brain/liver/lung) metastasis, chemotherapy, tumor grade, and sex that significantly influence survival outcomes of patients with chondrosarcoma. The development of decision tree models leveraging the C5.0 algorithm has yielded a promising prognostic tool for predicting overall survival. Recognizing the practical challenges of implementing graphical models in routine practice, we have taken a significant step forward by developing an interactive application and web‐based platform. This innovation simplifies the process of survival prediction, enabling clinicians to input patient‐specific information and receive immediate, visual feedback on survival probabilities.

### 4.1 Prognostic indicators for the survival statuses at various time points

The identification of pivotal prognostic indicators highlights the multifaceted nature of chondrosarcoma survival rates. These findings align with earlier studies that underscore the influence of these factors on patient prognosis.^12^, ^13^, ^14^, ^15^, ^16^, ^18^, ^19^, ^25^, ^26^, ^27^ The histological type and tumor grade of a chondrosarcoma reflect its biological behavior. Lower‐grade tumors grow more slowly and have less frequent local recurrence or distant metastasis, whereas higher‐grade tumors (particularly dedifferentiated types) exhibit greater malignancy and invasiveness, along with an increased risk of metastasis, leading to poorer overall survival.^12^, ^13^, ^14^, ^25^ Typically, for low‐grade chondrosarcomas, effective and safe treatment can be achieved through lesion curettage, while high‐grade tumors require more extensive surgical resection or even amputation.^12^, ^13^ Generally, patients who underwent complete surgical excision are in better overall performance status than those who did not receive surgery, resulting in higher overall survival rates.^14^, ^18^, ^19^ The age at diagnosis has also been confirmed as an independent prognostic factor for chondrosarcoma survival time in numerous studies,^14^, ^15^, ^16^, ^18^, ^26^ which may be due to older patients having poorer overall health and being more susceptible to becoming frail after developing the disease, as well as being unable to tolerate more extensive treatments. Patients with concurrent visceral (brain, liver, or lung) metastases indicate more advanced tumor progression, causing damage to other vital organs and negatively impacting overall treatment effectiveness, thus reducing their overall survival rate.^27^ Perioperative chemotherapy can achieve higher local control and reduce long‐term metastasis risks for certain higher‐grade tumors that are sensitive to chemotherapy, thereby extending survival times.^15^, ^18^, ^19^ Our study found that among Grade I chondrosarcoma patients aged between 65 and 76 years, female gender exerted a protective effect on their 120‐month survival status compared to males (Figure 8). We hypothesize that in this age group, women have relatively lower estrogen levels and correspondingly lower bone density, while lower‐grade tumors consist primarily of extracellular cartilaginous matrix with fewer cellular components, thus hindering the growth of tumor tissue. The impact of gender on the overall survival of patients with chondrosarcoma has also been corroborated in several prior studies.^18^, ^19^, ^26^

The temporal dynamics of prognostic factors in our study reveal a complex interplay between various clinical characteristics and their impact on survival outcomes at different stages of the disease. This finding challenges the conventional approach of assessing prognostic factors solely based on their overall effect across the entire follow‐up period. Our results suggest that the clinical utility of these factors may vary significantly depending on the temporal context in which they are considered. For instance, the identification of histological type and presence of visceral metastasis as potent predictors of short‐term (up to 24 months) survival underscores the immediate clinical relevance of these factors in the early management of patients. Clinicians may leverage this knowledge to prioritize interventions aimed at mitigating the acute risks associated with these conditions. Conversely, the emergence of tumor grade and sex as critical determinants of long‐term (≥60 months) survival highlights the need for sustained surveillance and tailored treatment strategies that account for the chronic implications of these factors. Moreover, the consistent influence of age at diagnosis and receipt of chemotherapy across all follow‐up intervals suggests that these variables play a foundational role in shaping the overall survival trajectory. Their ubiquitous impact emphasizes the importance of incorporating these factors into baseline risk assessments and treatment planning, regardless of the temporal focus of care.

These observations not only refine our understanding of the prognostic landscape but also have practical implications for personalized medicine. By recognizing the shifting significance of prognostic factors over time, healthcare providers can adapt their prognostic models and therapeutic approaches to reflect the changing needs of patients as their disease progresses. This adaptive approach stands in contrast to static prognostic models that fail to capture the dynamic nature of disease and patient responses, thereby potentially limiting the precision and effectiveness of clinical decision‐making. Therefore, our study contributes to the evolving paradigm of prognostication by illuminating the temporal specificity of prognostic factors. This nuanced perspective enhances the granularity of prognostic models and supports a more responsive and individualized approach to patient care, ultimately aiming to optimize survival outcomes and quality of life throughout the disease continuum.

### 4.2 Predictive systems established for survival estimation

Upon comprehensively and precisely screening for prognostic factors influencing the survival rates of chondrosarcoma, there is a need to further develop predictive systems that integrate all independent predictive elements for a more convenient estimation of patients' overall survival probabilities. Initially, clinicians typically utilize the TNM system, which assesses patients' initial survival status and risk stratification based on three key components: tumor characteristics (T), lymph nodes (N), and distant metastasis (M).^28^ Additionally, the AJCC staging system has been widely used for the prognostic evaluation of malignant tumors for a long time.^28^, ^29^ However, an increasing number of studies acknowledge the notable limitations of the AJCC system, as it only considers tumor size and histological metastasis.^30^, ^31^ Consequently, to conduct a more systematic and comprehensive assessment of chondrosarcoma prognosis, recent studies have selected prognostic factors for chondrosarcoma, integrating them with machine learning methods to develop predictive systems such as nomograms.^6^, ^14^, ^15^, ^16^, ^18^, ^19^, ^32^, ^33^ The modeling approaches employed in these studies often include Cox proportional hazards regression or logistic regression analyses, constructing the models as graphical prediction systems. Wu et al.^32^ established a nomogram model for predicting the overall survival of patients with limb chondrosarcomas, based on uni‐ and multi‐variable Cox regression. They identified that age, site, grade, tumor size, histology, stage, and use of surgery, radiotherapy, and chemotherapy are significantly associated with overall survival. Similarly, Sun et al.^16^ developed a nomogram to predict overall survival in patients with high‐grade chondrosarcoma, based on uni‐ and multivariate Cox analyses. In this study, age, histology type, tumor size, AJCC stage, regional expansion, and surgery were identified as independent prognostic factors. Many studies have also included CR‐ or MRI‐based radiomics in their prediction model establishment for predicting the clinical outcomes of chondrosarcomas.^21^, ^34^, ^35^, ^36^, ^37^ During the establishment of these models, however, all patients are analyzed as a single cohort, potentially obscuring unique and sensitive prognostic factors within specific subgroups of the cohort, making them difficult to uncover during the analytical process. In contrast, the decision tree model developed in this study utilizes the C5.0 algorithm and RPA, continuously segmenting the cohort based on the most significant predictive factor at each step. Throughout the development of the tree, the variables at each branch point are maintained as the most significant predictive factors, which facilitates the discovery of deeper interactions between variables and enhances the diagnostic accuracy of the model.^38^, ^39^ Furthermore, when users employ these previously established predictive systems for risk assessment, they must manually score each predictive factor and calculate the total score, which is then converted into corresponding survival probabilities for different time periods. This process remains somewhat complex, hindering the promotion of the model. To address the complexity and challenges associated with using the model, we have encapsulated the established predictive model into an app and deployed it on the server at *shinyapps.io* in this study. This app allows users to review the original datasets included in the analysis visually and customize explorations of the patient's epidemiological characteristics as needed. It also provides access to the entire process of model construction and validation. Most importantly, after selecting the clinical features of the patient to be predicted, the app automatically outputs the overall survival probabilities for 1, 2, 5, and 10 years, along with visual representations.

The findings of our study represent a pivotal stepping stone towards the development of a transformative tool that could redefine the landscape of clinical decision‐making and patient engagement. As we contemplate the translation of our research into a clinically applicable interactive application and web‐based platform, several critical considerations emerge. Firstly, the application must be designed with the end‐user in mind, ensuring that it is intuitive, informative, and empowering. Clinicians require a tool that simplifies complex data into actionable insights, thereby optimizing their decision‐making process. Patients, on the other hand, stand to benefit from a resource that elucidates the intricacies of their treatment options, fostering a greater understanding and encouraging active participation in their healthcare journey.

Key features of the envisioned application include personalized treatment protocols, interactive educational modules, clinical decision support tools, user feedback mechanisms, and stringent data security measures. These elements collectively aim to create a user‐centric platform that integrates seamlessly with existing clinical workflows and adheres to the highest standards of patient confidentiality. However, the path from conceptualization to implementation is fraught with challenges. It necessitates a multidisciplinary approach, engaging stakeholders from diverse backgrounds—researchers, clinicians, software developers, patient advocates, and legal experts—to ensure a holistic and effective solution. Pilot testing and phased rollouts are essential to evaluate the application's performance in real‐world scenarios, allowing for iterative refinements based on user feedback and empirical data.

### 4.3 Limitations

This study presents several limitations. Firstly, as a retrospective study, it is subject to potential biases in the data obtained, which may affect the validity of the results. To further verify the findings of this study, prospective clinical research is necessary. Secondly, there are inherent limitations associated with the SEER database itself, which may impact the generalizability of the conclusions of the study. The challenge of missing data is a well‐recognized issue that has significant implications for the robustness and interpretability of survival analyses. While traditional approaches to handling missingness, such as complete case analysis or imputation techniques, offer potential solutions, they each come with their own set of limitations. Complete case analysis, for instance, can lead to biased results if the missingness is nonrandom, and it reduces the sample size, potentially limiting the power of the study. On the other hand, imputation methods, especially those that rely on predictive models, may introduce spurious correlations and inflate the precision of estimates if the missing data are extensive. In light of these considerations, our study took another approach by incorporating the “unknown” category directly into the model framework. This strategy acknowledges the uncertainty associated with missing prognostic factors and avoids the pitfalls of assuming complete knowledge where none exists. By doing so, we aim to preserve the integrity of the dataset and provide a more realistic representation of the clinical setting, where prognostic information is not always fully available. However, the inclusion of an “unknown” category does present interpretational complexities. In the context of clinical decision‐making, the “unknown” category serves as a reminder of the need for prudence and the reliance on available evidence. Lastly, the survival outcome variable (survival time and status) was transformed into binary variables representing survival status at 12, 24, 60, and 120 months. Unlike the Cox model, the decision tree model used in this study does not handle censoring data as effectively. Consequently, censored data significantly influenced the accuracy of the results. To mitigate this issue, the censored records at the four time points were excluded from the analysis beforehand, leading to varying numbers of patients across the corresponding cohorts at each time point.

## 5 CONCLUSIONS

In conclusion, this investigation has robustly pinpointed pivotal prognostic indicators that markedly impact the survival prospects of individuals with chondrosarcoma. Through the amalgamation of these essential prognostic variables, sophisticated decision tree algorithms have been constructed, tailoring the estimation of survival to the individual level—a tool likely to be of immense value in shaping therapeutic plans and patient consultations. The empirical validation of these models confirms their high fidelity, evidenced by the close alignment between forecasted and observed survival outcomes, underscoring their potent predictive capabilities. To enhance user accessibility, we have introduced an interactive online application, streamlining the survival prediction process. This innovation empowers healthcare professionals to promptly enter patient‐specific data and receive instant, graphical representations of survival likelihoods, thereby optimizing the decision‐making experience.

## AUTHOR CONTRIBUTIONS

**Xiong‐Gang Yang:** Conceptualization (equal); data curation (equal); formal analysis (equal); writing – original draft (equal); writing – review and editing (equal). **Shan‐Shan Yang:** Conceptualization (equal); data curation (equal); formal analysis (equal); methodology (equal); writing – original draft (equal). **Yi Bao:** Methodology (equal). **Qi‐Yang Wang:** Methodology (equal). **Zhi Peng:** Writing – review and editing (equal). **Sheng Lu:** Supervision (lead).

## FUNDING INFORMATION

The study was supported by research grants from Project of Yunnan Key Laboratory of Digital Orthopedics (202005AG070004), Yunnan Orthopedics and Sports Rehabilitation Clinical Medical Research Center (202102AA310068), Yunnan Spinal Cord Disease Clinical Medical Center (ZX2022000101), Major Science and Technology Project of Yunnan Province Science and Technology Plan (202102AA310042), Yunnan Province “Ten Thousand People Plan” Famous Medical Project (YNWR‐MY‐2019‐058), Yunnan Medical Leading Talents Project (L‐2019006), and Social Development Project of Science and Technology Department of Yunnan Province (202,403 AC100003). The funders had no role in the design and execution of the study or writing of the manuscript.

## CONFLICT OF INTEREST STATEMENT

The authors declare that they have no competing interest.

## ETHICS STATEMENT

All procedures involving human participants were in accordance with the Helsinki declaration and its later amendments, and the use of de‐identified data from the SEER database exempted the study from requiring informed consent.

## Data Availability

The raw data is will be made available by the authors, without undue reservation. Further information can be available from the corresponding authors upon appropriate request.
